# Protective effect of heparin in the end organ ischemia/reperfusion injury of the lungs and heart

**DOI:** 10.1186/1749-8090-7-123

**Published:** 2012-11-15

**Authors:** Hikmet Selcuk Gedik, Kemal Korkmaz, Havva Erdem, Evvah Karakilic, Gokhan Lafci, Handan Ankarali

**Affiliations:** 1 Cardiovascular Surgery Department of Ankara Numune Education and Research Hospital, Talatpasa Bulvari, 06100, Ankara, Turkey; 2 Cardiovascular Surgery Department of Ankara Numune Education and Research Hospital, Talatpasa Bulvari, 06100, Ankara, Turkey; 3 Pathology Department of Duzce University School of Medicine, Konuralp, Duzce, Turkey; 4 Emergency Department of Ankara Numune Education and Research Hospital, Talatpasa Bulvari, 06100, Ankara, Turkey; 5 Cardiovascular Surgery Department of Turkiye Yuksek Ihtisas Hospital Sihhiye, 06100, Ankara, Turkey; 6 Biostatistic Department of Duzce University School of Medicine, Konuralp, Duzce, Turkey

## Abstract

**Background:**

Ischemia/reperfusion (I/R) injury is harmful to the cardiovascular system and is responsible for the inflammatory response and multiple organ dysfunctions. In this study we investigated the effect of activated clotting time level on the aortic cross-clamping triggers a systemic inflammatory response and it effects to lungs and heart.

**Methods:**

End organ concentrations of interleukin-6 (IL-6), myeloperoxidase (MPO) and heat shock protein 70 (HSP-70) were determined in four groups of Spraque Dawley rats: ischemic control (operation with cross clamping received IP of 0.9% saline at 2 ml/kg n=7) Sham (operation without cross clamping, n=7), heparin (ACT level about 200), High dose heparin (ACT level up to 600) The infrarenal aorta was clamped for 45 minutes by a mini cross clamp approximately 1cm below the renal artery and 1cm iliac bifurcation in all groups without sham group. Heparin was given intraperitoneal (IP) before the procedure. All rats were sacrificed 48 h later. In a second experiment, the effects of I/R on remote organs (lungs and heart) were harvested for analysis. We evaluated tissue levels of myeloperoxidase, interleukin-6, and heat shock protein (HSP-70) were analyzed as markers oxidative stress and inflammation. Histological analyses of the organs were performed.

**Results:**

The lungs paranchymal MPO and HSP-70 levels significantly decreased (p<0.05), but IL-6 level was not significant (p>0.05) in heparinized and high dose heparinized groups when compared to ischemic control group. Histopathological evaluation as edema, cell degeneration, inflammation statistically significantly decreased in both group heparinized and high dose heparinized compared with ischemic control group (p<0.05). The heart paranchymal MPO levels significantly decreased in heparinized and high dose heparinized groups when compared to ischemic control group (p=0.023). IL-6, HSP-70 levels were not significant heparinized and high dose heparinized groups when compared to ischemic control group (p=0.0489, p=0.0143). Histopathological evaluation as degeneration statistically significantly decreased in both group heparinized and High dose heparinized compared with ischemic control group (p=0.005).

**Conclusion:**

Heparin decreased remote organs injury on the lung and heart after ischemia/reperfusion of infra-renal section of the body in the rat model. So, we should be balance to act level for avoid to I/R injury per operative and early post operative period as providing ACT level nearly 200.

## Background

Thoraco-abdominal aortic surgery can cause Ischemia/Reperfusion I/R injury in not only the spinal cord, but also in the remote organs such as lungs and heart during the operations [1].

It can result in mortality and morbidity because of remote organ injury in early post-operative period. Despite the exact pathophysiologic mechanism that underlie ischemia reperfusion of the lower body sustain to be defined inflammatory response is known to play an important role after ischemia subscribes to remote organ injury [2,3].

Cytokines such as IL-6 are important mediators of the inflammatory response in ischemia. IL-6 is released in response to infection, burns, trauma and neoplasia and its functions range from key roles in acute phase protein induction to B and T cell growth and differentiation [4]. The cellular stress response can mediate cellular protection trough expression of heat shock protein (HSP-70), which can interfere with the process of apoptotic cell death [5-7].

Myeloperoxydase (MPO) is one of the distinct indicators for the tissue infiltration of neutrophilic granulocytes. MPO activity which is response to the ischemia reperfusion injury increases in the end organ tissue substantially [8].

The objective of our study is to prevent thrombus formation in the microcirculation by monitoring the effectiveness of heparin, in this way to demonstrate how much we could suppress inflammation occurring after ischemia / reperfusion by limiting ischemia.

## Method

### Animal and surgical procedure

This study was approved by the Animal Experimental Committee of Duzce University Graduate School of Medicine Animal care and all procedures were performed according to guide for the care and use the National Institute of Heat’s. Rats weighed between 250 and 350 gr and a total of 28 male Spraque-Dawley rats were used for the experiment. The animals were fed a standard rat chow using the same nutrition protocol and were then studied. None of them had any neurological disorders before operation. Hemochron Jr signature plus (Keller Medical GmbH, Bad Soden, Germany) was used to detect ACT level.

### Study groups

Animals were divided into four groups. Sham group (n=7) was appointed as Group-1. The operation was performed in the same way, but without aortic occlusion and heparin administration. Only laparatomy was performed in this group. Non- heparinized control group (n=7) was appointed as Group 2. In this group animals’ abdominal aortas were clamped for 45 minutes by an aneurysm clip, approximately 1cm below renal arteries and 1cm above the iliac bifurcation. Heparin treated group (n=7) was appointed as Group 3. 400 IU/kg of heparin was administered via intraperitoneal to all animals immediately before the procedure. High dose heparin treated group (n=7) was appointed as Group-4. In this group heparin dose was increased 800 IU/kg for ACT level of 600 sec immediately before the procedure. All cross-clamped groups were reperfused after spinal cord ischemia.

### Operative procedure and technique

Rats were premedicated with ketamine (50 mg/kg) and xylazine (5 mg/kg) intraperitoneally. The maintenance of anesthesia was established with intermittent delivery of ketamine, without endotracheal intubation and mechanical ventilation. Intraperitoneal cephalosporine (10 mg/kg) was administered before skin incision. Postoperative analgesia was provided with tramadol per 12 hours. Temperature probe was inserted into the rectum.

After the surface cleaning of the surgical area and standard midline laparatomy, the abdominal aorta was explored trough a transperitoneal approach retracting the intestines. The abdominal aorta was clamped with mini aneurysm clamp between below renal arteries above the iliac bifurcation. Temperature was maintained between 36,5 and 37,5°C during the spinal procedure with heating lamp. Artery blood pressure and heartbeat were monitored continuously the left carotid artery cannula.

After administrating study protocol in all rats, aortic clamps were removed and anterior abdominal wall was sutured by using 5/0 polyprolene suture.

At 48^th^ hour the all animals were anesthetized with penthobarbital (20 mmg/kg) and sacrificed. The lungs and heart were dissected totally and fixed in buffered formalin for 7 days.

### Experiment-1: inflammatory markers production

In this experiment the production of the inflammatory markers IL-6, HSP-70, MPO were studied in four groups of animals. Blood samples were obtained at the end of 48 h. At the time of blood sampling the chest wall was cleansed with cholorohexidine in spirit, and a sterile 10ml syringe was then used to obtain a blood sample by direct cardiac puncture. Blood samples for cytokine assay were collected into heparinized (20 unit/ml blood) sterile tubes and immediately transferred on ice to be centrifuged at 2000 rpm (at 4°C) for 10 minutes and stored (−70°C) until the time of assay for IL-6, MPO and HSP-70.These markers level obtained not only from blood samples but also from lungs and heart tissue.

### Experiment-2: Histopahological Assessment

A midline laparotomy was performed to all animals immediately following blood sampling and total lobe of the lung and heart were removed and fixed in 10% formalin for histopathologic examination. Paraffin sections (4 μm thickness) were prepared. The slides were evaluated under light microscopy (Olympus BX51; Olympus Corp., Tokyo, Japan) at 400x magnification. 5 μm thick sections were placed on polylysine-coated slides and stained with hematoxylin and eosin (H & E).

#### Immunohistochemistry

##### Analysis of HSP

Paraffin sections (4 μm thick) were prepared. Tissue sections were deparaffinized and hydrated in xylenes and graded alcohol. The sections were incubated with primary anti-HSP70 (clone BRM.22, dilution 1/80, Biogenex, San Ramon, California) diluted in buffer. PBS was used as negative control.

#### Analysis of IL-6

The polyclonal anti-human IL-6 receptor antibody C-20 (Santa Cruz Biotechnologies, Santa Cruz, CA, USA) was used for the immunohistochemical detection of IL-6 receptor. This antibody was diluted 1: 20. IL-6 receptor immunostaining was also performed according to a streptavidin-biotin-peroxidase protocol. The secondary anti-rabbit antibody was diluted 1: 500. Negative controls were performed by omitting the first antibody.

#### Analysis of MPO

The lung and heart tissue MPO activities were evaluated immuno-histochemically using an anti-MPO kit according to the manufacturer’s protocol. Briefly, samples on polylysine-coated slides were deparaffinized and rehydrated. Then, the microwave antigen retrieval procedure was performed, and the samples were incubated in a 3% H_2_O_2_ solution to inhibit endogenous peroxidase. To block nonspecific background staining, the sections were incubated with a blocking solution. Then the sections were incubated with primary anti-MPO antibody, followed by incubation with biotinylated goat anti-mouse antibody. After incubating with the chromogenic substrate (DAB), the sections were counterstained with hematoxylin and eosin (H & E).

The slides were examined under a light microscope and all analyses were performed by two pathologists blinded to the group assignments. The staining of cytoplasmic MPO in the neutrophils was evaluated, and the results were expressed as the percentage of neutrophils cytoplasmically stained positive for MPO.

Tissues with no evidence of staining, or only rare scattered positive cells, less than 3%, were recorded as negative. The immunohistochemical results were evaluated for intensity and frequency of staining. The intensity of staining was graded as 0 (negative), 1 (weak), 2 (moderate), and 3 (strong). The frequency was graded from 0 to 4 by the percentage of positive cells as follows: grade 0, <3 %; grade 1, 3-25%; grade 2, 25-50%; grade 3, 50-75%; grade 4, more than 75%. The index score was the product of multiplication of the intensity and frequency grades, which was then classified into a 4 point scale: index score 0 = product of 0, index score 1 = products 1 and 2, index score 2 = products 3 and 4, index score 3 = products 6 through 12.

### Statistical analysis

Statistical analysis and calculations were performed by using SPSS 15 for Windows (Chicago,IL). Results were expressed as the mean (standard error mean). Kruskal-Wallis analysis of variance was used to detect differences between groups and statistical comparisons were made using the Mann–Whitney U test. A *p* value of < .01 was considered statistically significant. A *p* value of < .05 was considered statistically less significant.

## Results

There was no significant difference in terms of body temperature and arterial blood pressure.

### Histopathological and immunohistochemical evaluation

**Figure 1:**
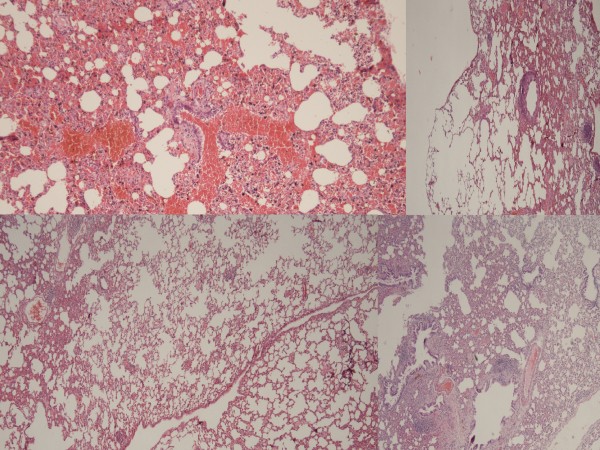
**H & E x100 staining of the lung tissue.** Left abobe; control group with intense cellular degeneration, edema, inflammation and congestion. Left-below: High dose heparin group was similar with the sham group (right below). The injury was moderate in the heparin group (right above).

The lung tissue was evaluated. Ischemic control group of the examination compared with sham group showed more edema, inflammation and cell degeneration according to histopathologic evaluation. Edema, inflammation and cellular degeneration differed significantly between the study groups (p value 0.032, 0.012 and < 0.0001, respectively). However level of congestion was similar (p=0.124) Therefore there was significantly more edema, inflammation and cellular degeneration in the ischemic control group (Table 1 and Figure 1).

**Table 1:** Histopathologic results of the lung tissue

**H&E**	**Grade**	**Ischemic control**		**Sham**		**Heparinized**		**High dose-heparinized**		***P values***
		**Number**	**%**	**Number**	**%**	**Number**	**%**	**Number**	**%**	
Edema	0	0	0	3	42.9	4	57.1	4	57.1	0.032
	1	7	71.4	4	57.1	3	42.9	3	42.9	
Inflammation	1	4	57.1	7	100	6	85.7	7	100	0.012
	2	3	42.9	0	0	1	14.3	0	0	
Cell Degeneration	0	0	0	0	0	0	0	1	14.3	<0.0001
	1	0	0	7	100	6	85.7	6	85.7	
	2	6	85.7	0	0	1	14.3	0	0	
	3	1	14.3	0	0	0	0	0	0	
Congestion	0	0	0	1	14.3	0	0	0	0	0.124
	1	3	42.9	6	85.7	6	85.7	7	100	
	2	3	42.9	0	0	1	14.3	0	0	
	3	1	14.3	0	0	0	0	0	0	

**Figure 2:**
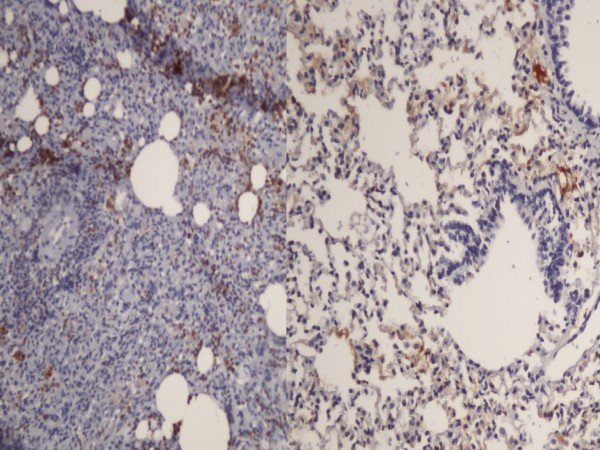
**Left panel: MPO staining was intense in the control group.** Right panel: Less intense staining in the high dose heparin group (MPOX200).

**Figure 3:**
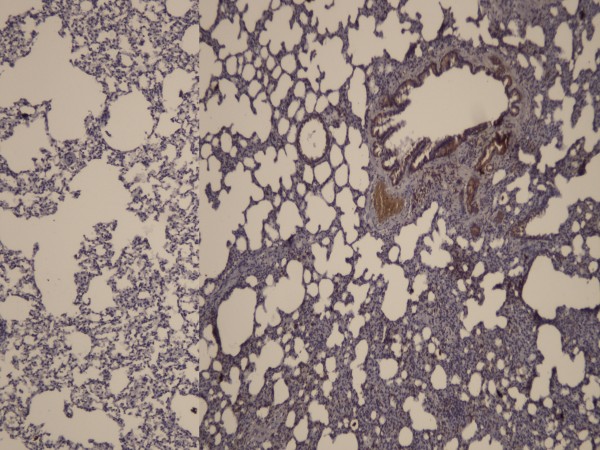
Staining with HSP-70 was stronger in the control group (Right panel) than the high dose heparin group (HSP-70X200).

Immunohistochemical markers as, MPO, HSP-70 and IL-6 parameters were studied from the lung tissue samples. Statistical difference was found among the groups at 48 hours postoperatively with MPO and hsp-70 stains (p value 0.019 and 0.05, respectively), whereas there was no difference among the groups with IL-6 stain (p=0.089) (Figure 2). Significant difference was seen in the Sham group when compared with the other groups. Intensity of staining with MPO was greatest in the ischemic control group followed by heparin group. Also, control group showed the most intense staining with Hsp-70 (Table 2) (Figure 3). The lowest HSP-70 level was measured in the sham group, after heparinized groups while the highest HSP-70 level was measured in the ischemic control group.

**Table 2:** Immunohistochemical results of the lung tissue

	**Grade**	**Ischemic control**		**Sham**		**heparinized**		**High dose heparinized**		***P value***
		**Number**	**%**	**Number**	**%**	**Number**	**%**	**Number**	**%**	
MPO	0	0	0	4	57.1	2	28.6	5	71.4	0.019
	1	5	71.4	3	42.9	5	71.4	2	28.6	
	2	2	28.6	0	0	0	0	0	0	
HSP	0	0	0	4	57.1	2	28.6	2	28.6	0.050
	1	5	71.4	3	42.9	5	71.4	5	71.4	
	2	2	28.6	0	0	0	0	0	0	
IL-6	0	0	0	4	57.1	2	28.6	4	57.1	0.089
	1	6	85.7	3	42.9	5	71.4	3	42.9	
	2	1	14.3	0	0	0	0	0	0	

**Figure 4:**
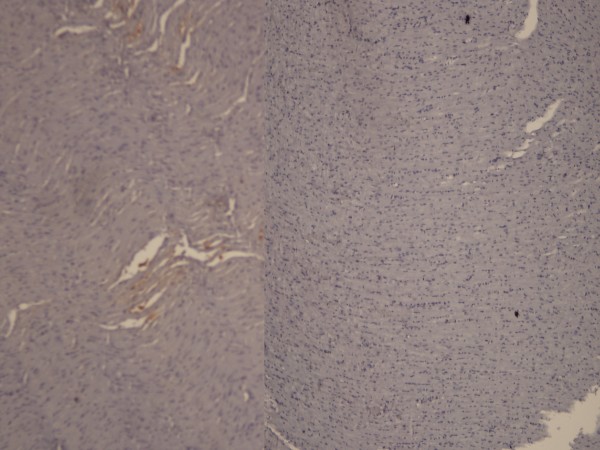
IL-6 staining (left panel) and mild staining with HSP-70(right panel) in the high dose heparin group (X200).

Examination of the heart tissue revealed that ischemic control group showed more degeneration according to histopathologic evaluation (p=0.005); whereas there was no difference between the groups according to the level of edema, inflammation and congestion (p values, 0.613, 0.499 and 0.558, respectively (Figure 4). Degeneration was worst in the ischemic control group followed by heparin and high dose-heparin groups (Table 3).

**Table 3:** Histopathological results of the heart tissue

	**Grade**	**High dose-heparinized**		**Sham**		**İschemic control**		**Heparinized**		***P value***
		**Number**	**%**	**Number**	**%**	**Number**	**%**	**Number**	**%**	
Edema	0	4	57.1	5	71.4	5	71.49	5	71.4	0.613
	1	3	42.9	2	28.6	2	28.6	2	28.6	
Inflammation	0	3	42.9	5	71.4	3	42.9	5	71.4	0.499
	1	4	57.1	2	28.6	4	57.1	2	28.6	
Degeneration	0	4	57.1	6	85.7	0	0	1	14.3	0.005
	1	3	42.9	1	14.3	6	85.7	6	85.7	
	2	0	0	0	0	1	14.3	0	0	
Congession	0	4	57.1	5	71.4	6	85.7	6	85.7	0.558
	1	3	42.9	2	28.6	1	14.3	1	14.3	

**Figure 5:**
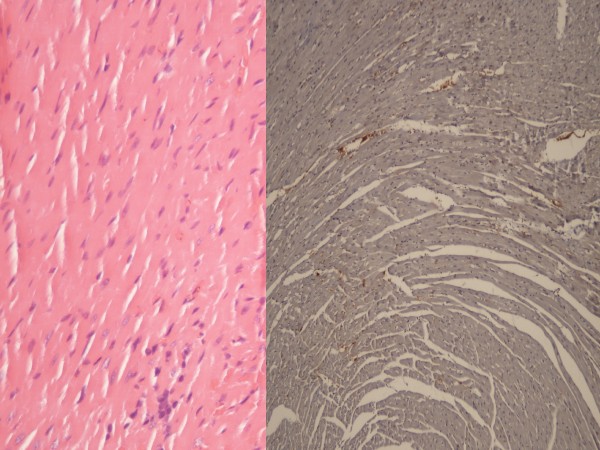
**Mild degeneration, congestion and several inflammatory cells in the heart tissue of the control group (left panel).** Moderate staining with MPO (right panel) (X200).

Comparison of immunohistochemical markers of the heart tissue revealed that there was a significant difference with MPO staining (p=0.023) which was strongest in the control group, where as IL-6 and Hsp-70 levels did not differ among the groups (p values, 0.483 and 0.4 respectively) (Figure 5).

**Figure 6:**
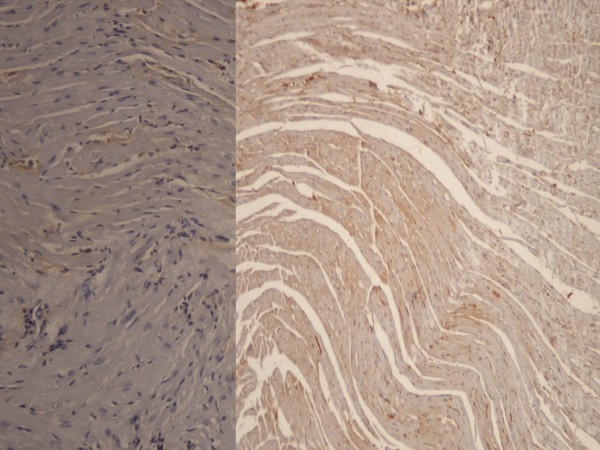
Moderate staining with IL-6 (left panel) and HSP-70 (right panel) in the heart tissue of the control group (X200).

Grade 1 and 2 staining with HSP-70 was strongest in the control group (n=4 and n=1, respectively) where as it was weakest in the high dose heparinized group. Grade 1 staining was strongest with IL-6 in the control group (n=6) where as it was again weakest in the high dose heparinized group (Figure 6) These qualitative differences did not reach statistical significance possibly due to the low number of samples (Table 4).

**Table 4:** Immunohistochemical results of the heart tissue

	**Grade**	**Ischemic control**		**Sham**		**Heparinized**		**High dose-heparinized**		***P value***
		**Number**	**%**	**Number**	**%**	**Number**	**%**	**Number**	**%**	
MPO	0	0	0	4	57.1	4	57.1	4	57.1	0.023
	1	7	100	3	42.9	3	42.9	3	42.9	
HSP	0	2	28.6	5	71.4	4	57.1	5	71.4	0.489
	1	4	57.1	2	28.6	3	42.9	2	28.6	
	2	1	14.3	0	0	0	0	0	0	
IL-6	0	1	14.3	4	57.1	3	42.9	5	71.4	0.143
	1	6	85.7	3	42.9	4	57.1	2	28.6	

## Discussion

Thoracoabdominal aortic surgery can cause systemic inflammatory response and ischemia occurs when the blood flow through the major arteries that supply blood to remote organs slowed or stopped [9,10]. So, microthrombus formation associated with blood flow slowing or stopping causes widespread inflammation and severe ischemia. This process causes of multiorgan disorders and associates with histopathological and functional changes [11,12]. Thoracoabdominal aortic clamping can cause transient ischemia may be an inevitable consequence of descending or thoracoabdominal aortic surgery due to microthrombus formation for inadequate heparin dose. Especially, when the hematocrit level is higher than 40 mg/dl microthrombus may easily form. It was suggested that microcirculatory disturbances such as higher blood viscosity due to hemoconcentration and microthrombus formation were related to the ischemia [13]. Heparin has beneficial effects on organs injury in the events due to its anticoagulant activity [14].

The present study showed that levels of HSP-70, IL-6 and MPO at the tissue level after ischemia/reperfusion were increased. Intensity of edema, cellular degeneration and congestion in the lung tissue after I/R was decreased after heparin treatment and the histopathologic protective effect of heparin was shown in the present study. Myeloperoksidaz and IL-6 are acute phase proteins and increases with inflammation [15,16]. HSP-70 is a well known cytoprotective agent and increases after I/R and its level is directly related with the degree of tissue injury and inflammation. In the present study, HSP-70 level was highest in the control group, followed by heparin, high dose- heparin and sham groups.

Heat shock protein 70 (Hsp70) has been shown to have an anti-apoptotic function, but its mechanism is not clear in heart. Histopathologic changes were mild in heart. Only cellular degeneration showed significant difference among groups after I/R [17,18]. The changes in IL-6 and HSP-70 should be studied in larger cohorts.

Lower levels of MPO in heart and lung tissue after I/R in the heparin ve high dose heparin groups, showed that the inflammation and injury was significantly lower with this treatment. The decrease in HSP-70 in these groups also was an indirect proof of lower tissue injury and better cellular protection. Markers of injury and repair were analyzed together in the present study.

The half-life of heparin increases from approximately 30 min following an IV bolus of 25 U/kg, to 60 min with a bolus of 100 U/kg, and to 150 min with a bolus of 400 U/kg [19]. We administer heparin dose 100 u/kg bolus and maintain ACT level as 200 in abdominal aortic surgery, routinely. In the present study there was no significant difference between heparin groups (ACT: 200 and ACT: 600) in terms of histopathologic changes or biochemical inflammatory response in the lungs and heart. Also, clinical pattern was not different between the groups. The HSP-70 expression was significantly higher and MPO activity was significantly lower in ischemic control group compared with heparin groups.

## Conclusion

ACT levels of 200, as used in the real-world clinical practice, should be maintained in the open heart surgery. No beneficial effects were seen with the use of higher levels of ACT in the present study. Taking into account that, bleeding complications will be much less the target dose of heparin should be maintained at this level in daily practice.

## Competing interests

The authors declare that they have no competing interests.

## Authors’ contributions

HSG, GL, KK carried out the animal study. EK carried out the sequence alignment and drafted the manuscript. HE carried out the immunohistochemical and pathological study. HA carried out the statistical analysis. All authors have read and approved the final manuscript.
